# Bioinspired Perovskite Nanocrystals‐Integrated Photonic Crystal Microsphere Arrays for Information Security

**DOI:** 10.1002/advs.202105278

**Published:** 2022-01-20

**Authors:** Feika Bian, Lingyu Sun, Hanxu Chen, Yu Wang, Li Wang, Luoran Shang, Yuanjin Zhao

**Affiliations:** ^1^ Department of Clinical Laboratory Institute of Translational Medicine The Affiliated Drum Tower Hospital of Nanjing University Medical School Nanjing 210008 China; ^2^ State Key Laboratory of Bioelectronics School of Biological Science and Medical Engineering Southeast University Nanjing 210096 China; ^3^ Shanghai Xuhui Central Hospital Zhongshan‐Xuhui Hospital and the Shanghai Key Laboratory of Medical Epigenetics International Co‐laboratory of Medical Epigenetics and Metabolism (Ministry of Science and Technology Institutes of Biomedical Sciences) Fudan University Shanghai 200433 China; ^4^ Oujiang Laboratory (Zhejiang Lab for Regenerative Medicine, Vision and Brain Health) Wenzhou Institute University of Chinese Academy of Sciences Wenzhou 325001 China

**Keywords:** bioinspired, hydrochromic, information security, perovskite nanocrystal, photonic crystal, structural color

## Abstract

Information security occupies an important position in the era of big data. Attempts to improve the security performance tend to impart them with more additional encryption strategies. Herein, inspired by the wettability feature of *Stenocara* beetle elytra and signal model of traffic light, a novel array of perovskite nanocrystals (PNs)‐integrated PhC microsphere for information security is presented. The photoluminescent PNs are encapsulated in angle‐independent PhC microspheres to impart them with binary optical signals as coding information. Through the multimask superposition approach, PNs‐integrated PhC microspheres with different codes are placed into fluorosilane‐treated PDMS substrate to form different arrays. These arrays could converge moisture on PhC microspheres in wet environment, which avoids the ions loss of the PNs and effectively prevented mutual contamination. In addition, the fluorescence of the PNs inside PhC microspheres could reversibly quench or recover in response to the environmental moisture. Based on these features, it is demonstrated that the PNs‐integrated PhC microsphere arrays could realize various information encryption modes, which indicate their excellent values in information security fields.

## Graphical abstract

Novel perovskite nanocrystals (PNs)‐integrated photonic crystals (PhC) microsphere arrays inspired by *Stenocara* beetle and traffic light are presented for information security. The photoluminescent PNs are encapsulated in angle‐independent PhC microspheres to impart them with binary optical signals as coding information. The PNs‐integrated PhC microsphere arrays could realize various information encryption modes, which indicate their excellent values in information security fields.

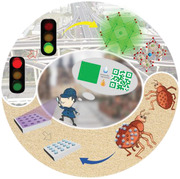

## 1 Introduction

Information security has attracted much attention since ancient times.^[^ ^1^, ^2^, ^3^, ^4^, ^5^, ^6^ ^]^ The methods of information protection have gradually developed from sealing wax, invisible ink to steganography, coding, etc. Information coding is a strategy that stores and transmits message by converting to codes after reasonable design, which has been widely used in the field of communication and anticounterfeiting.^[^ ^7^, ^8^, ^9^, ^10^ ^]^ Among various information coding strategies, photonic crystals (PhCs) that are composed of periodically arranged nanoparticles are attracting great attention due to their stable optical properties.^[^ ^11^, ^12^, ^13^, ^14^, ^15^, ^16^, ^17^ ^]^ Photonic bandgaps (PBGs) arisen from these ordered structures endow PhC codes with characteristic reflection peaks for applications in various fields including multiplexed detection, anti‐counterfeiting, and so on.^[^ ^18^, ^19^, ^20^, ^21^, ^22^, ^23^ ^]^ Despite with many progresses, the majority of PhCs utilized for information coding are angle dependent, which would cause inconsistence of the obtained message from different observing angles.^[^ ^24^ ^]^ Besides, most of the information hidden by PhCs could be easily distinguished through observing structural colors and analyzing reflection spectra, which may lead to information leakage. Therefore, a new PhC information coding strategy with properties of consistent encryption and anticounterfeiting is still highly anticipated.

**Figure 1:**
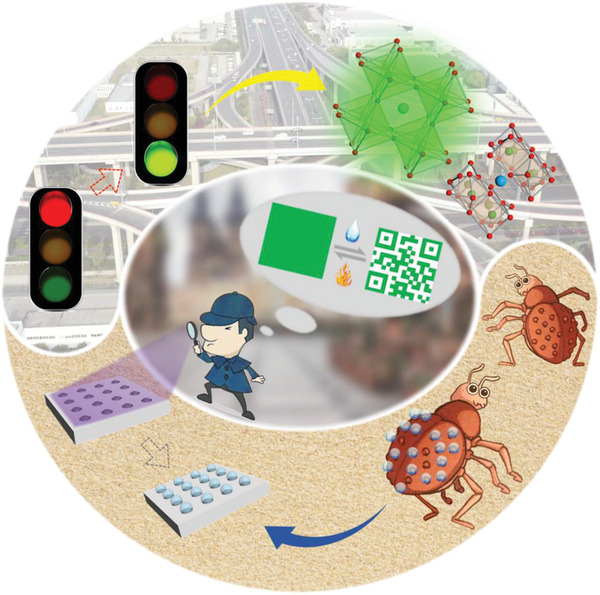
Schematic illustration of the bioinspired PNs‐integrated PhC microsphere arrays for information security. The PhC microsphere arrays have a *Stenocara* beetle elytra‐inspired wettability structure. The photoluminescent PNs inside PhC microspheres could reversibly quench or recover in response to the moisture, which mimics the signal model of traffic light.

In this paper, inspired by the wettability feature of *Stenocara* beetle elytra and signal model of traffic light, we present a novel array of perovskite nanocrystals (PNs)‐integrated PhC microsphere for information security, as schemed in **Figure** **1**. In nature, the hydrophobic elytra of *Stenocara* beetle exists plenty of hydrophilic bumps to collect water from air for its survival in the desert.^[^ ^25^, ^26^, ^27^ ^]^ Inspired by this, we have previously demonstrated that a hydrophilic PhC array in a hydrophobic substrate could possess the similar wettability feature.^[^ ^28^ ^]^ Correspondingly, PNs are emerging photoelectric material with advantages of extraordinary physicochemical properties, high photoluminescence quantum yield (PLQY), and low cost.^[^ ^29^, ^30^, ^31^, ^32^, ^33^, ^34^ ^]^ Remarkably, the fluorescence of PNs is water sensitive owing to their molecular conformation change in wet condition similar to the traffic light.^[^ ^35^, ^36^, ^37^, ^38^ ^]^ Moreover, benefitting from the spherical symmetry, PhC microsphere units possess angle‐independent constant reflection peaks for serving as coding signals.^[^ ^39^, ^40^ ^]^ Therefore, it would be a novel strategy for information coding by imitating the wettability feature of *Stenocara* beetle, the signal model of traffic light, and integrating PNs into spherical PhCs, which is expected to open a new chapter in information security fields.

Herein, we employed a multimask superposition approach for fabricating an array composed of PN‐integrated PhC microspheres by immobilizing hydrophilic PNs‐integrated PhC microspheres into fluorosilane‐treated hydrophobic polydimethylsiloxane (PDMS) substrate. The PNs‐integrated PhC microspheres were generated by simply soaking and drying perovskite solution in their gaps. We confirmed that the PNs‐integrated PhC microspheres could maintain both photoluminescence of PNs and structural color of PhCs without mutual destruction. Thus, the composite microspheres could provide binary optical coding. With the assistance of well‐designed masks, these microspheres with different coding information could be fixed in setting positions to form the array according to requirements. The resultant array with *Stenocara* beetle‐inspired wettability structure could converge moisture on PhC microspheres in wet environment, which avoided the ions loss of the PNs and effectively prevented mutual contamination. In addition, the fluorescence of the PNs inside PhC microspheres could reversibly quench or recover in response to the moisture of environments. Based on these features, we have demonstrated the practical values of the PNs‐integrated PhC microsphere arrays in different information encryption modes. These results indicate that the PNs‐integrated PhC microsphere arrays are a promising approach for information security applications.

## 2 Result and Discussion

In a typical experiment, the photonic crystals (PhCs) microspheres were self‐assembled in a capillary microfluidic device by silica nanoparticles with uniform diameter (Figure S1, Supporting Information). By adjusting the flow rates of dispersed phase containing SiO_2_ nanoparticles, different sizes of PhC microspheres could be generated. Then the PhC microspheres would be calcined to make the silica nanoparticles’ surface fuse and bond, which could enhance the mechanical strength of the whole microsphere. These PhC microspheres were immersed in the DMF solution dissolved with CsPbBr_2_I and then taken out when they were completely infiltrated. Owing to the existence of capillary force, CsPbBr_2_I solution still existed in the gaps inside PhC microsphere. With the volatilization of DMF, CsPbBr_2_I wound gradually precipitate into nanoscale crystals because of the confined space (Figure S2, Supporting Information). The CsPbBr_2_I nanocrystals could impart the PhC microspheres with fluorescent property under ultraviolet light. By repeating the above procedures using different solutions, various PhC microspheres encapsulated with different PNs were collected for the subsequent experiment.

**Figure 2:**
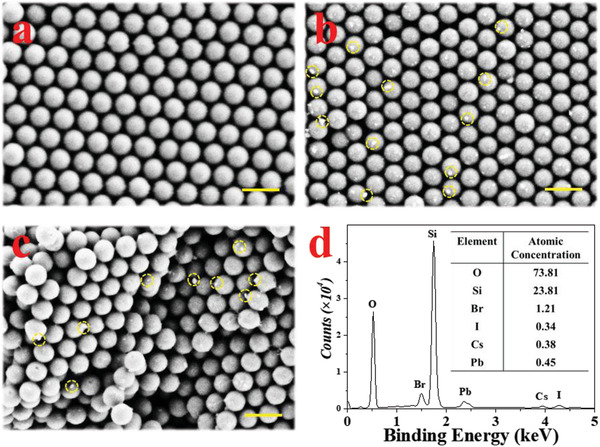
FESEM images of a) PhC microsphere with smooth surface; b) PhC microsphere after decorated with 0.1 m CsPbBr_2_I, some white crystals could be obseverd. c) The internal microstructure of PNs‐integrated PhC microsphere, also have crystals in the microsphere. d) Energy dispersive spectrometer energy spectrum of PNs‐integrated PhC microsphere. Scale bars are 500 nm.

In order to further analyze the microstructure, a field emission scanning electron microscopy (FESEM) was utilized to investigate the PNs‐integrated PhC microspheres. It was found that SiO_2_ nanoparticles assembled into a closely packed hexagonal ordered morphology, as shown in **Figure** **2**a. After PNs decorating, CsPbBr_2_I nanocrystals were observed among the voids of SiO_2_ nanoparticles (Figure 2b). Because of the limited space, the overgrowth of CsPbBr_2_I was limited and finally formed nanocrystals. In addition, the amount of CsPbBr_2_I nanocrystals increased with the promoting concentration of perovskite DMF solution's (Figure S3a,b, Supporting Information). Then the PhC microspheres were cut into hemispheres to characterize the internal structure (Figure S3c, Supporting Information). FESEM image showed that the SiO_2_ nanoparticles in PhC microsphere were also ordered as closely packing, and there also existed distinct CsPbBr_2_I nanocrystals among the interstices in PhC microspheres (Figure 2c). Subsequently, the components of PNs‐integrated PhC microsphere were analyzed by energy dispersive spectrometer (EDS) energy spectrum. The EDS data displayed that the composite microspheres not only contained the constituent elements of PhC microspheres, but also possessed the components of CsPbBr_2_I nanocrystals appearing in the image (Figure 2d). These results indicated that PNs could be easily integrated into the interior of PhC microspheres through this simple method.

It was worth nothing that the bare PhC microspheres were assembly of colloidal nanoparticles with a 3D periodic face‐centered cubic arrangement, which is a typical PhC material (Figure S3d, Supporting Information). Owing to the hexagonally ordered closely packed nanostructure of the PhC microsphere, these microspheres were endowed with distinct structural color with a Bragg reflection peak tunable within the visible range which were generated by photonic bandgap (PBG). The characteristic reflection peak could be expressed by the Bragg diffraction formula

(1) $$m\;\lambda_{0}=\;2dn_{\mathrm{average}}\sin\theta$$

Since the (111) plane of the crystal is densely packed structure and the observation angle is perpendicular to the PhC, making us only explore the first‐order diffraction and the case where sin *θ* (*θ* = 90°) is equal to 1. Thus, formula (1) can be simplified to^[^ ^18^ ^]^

(2) $$\lambda_{0}=\;1.633dn_{\mathrm{average}}$$

In this reduced Bragg's equation, the characteristic reflection peak corresponding to the coding structural color of the PhC microspheres can be expressed by *λ*_0_. This property gave rise to the structural color of the microspheres, which served as units for creating the anticounterfeiting arrays. The crystalline interplanar spacing *d* means the distance between the centers of adjacent SiO_2_ nanoparticles in the closely packed microstructure. Owing to the different materials, the refractive index (*n*_average_) of the whole PhC microsphere could be calculated by averaging the refractive index of SiO_2_ nanoparticles and the medium in their gaps according to volume fraction. Therefore, the factor affecting the characteristic reflection peak of PhC microspheres is only the distance between neighboring SiO_2_ nanoparticles’ centers, that is, the size of these nanoparticles utilized to fabricate PhC microspheres. Besides, the structural color and the corresponding reflection peak of PhC microspheres remained constant at different glancing angles benefiting from the spherical symmetry, while that of anisotropic shapes, for example, PhC films or bulk PhC materials, had an obvious shift. This characteristic endows the PhC microsphere with a stable optical signal as information coding.

**Figure 3:**
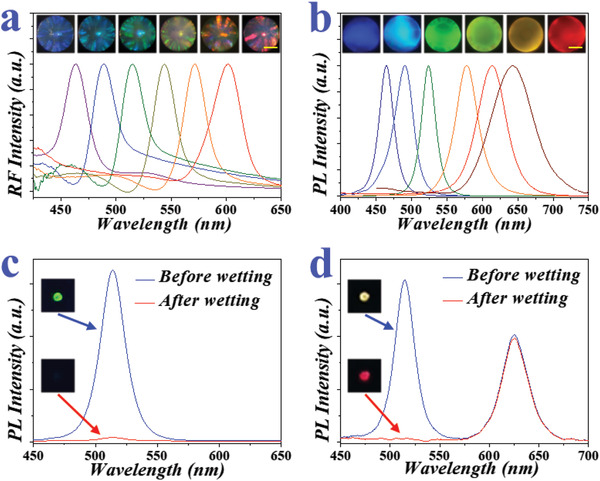
a) Optical images and reflection spectra of six different PhC arrays, scale bar is 100 µm. b) Fluorescent images and emission spectra of six different PNs‐integrated PhC microspheres, scale bar is 100 µm. c) Emission spectra and fluorescent images of the green PNs‐integrated PhC microspheres before and after wetting. d) Emission spectra and fluorescent images of the red QDs/green PNs‐integrated PhC microspheres before and after wetting.

We speculated that the presence of PNs would not destruct the structural color of the composite PhC microspheres, given that their periodically ordered structure was maintained (Figure 2c). To test this, a fiber optic spectrometer was employed to explore their optical performance. According to simplified Bragg diffraction formula, SiO_2_ nanoparticles with different sizes were utilized to generate six kinds of PhC microspheres with corresponding characteristic reflection peaks (**Figure** **3**a). Compared with dyed mesoporous silica microspheres or other types of luminescent beads, the structural color of the PhC microspheres is mainly reflected in the central spot. Thus, information encryption based on PhC microspheres is more covert since they are relatively hidden in large scale. In addition, the reflection signal of the PhC microspheres is more stable than dyes or pigments since it is structurally based. After decorated with high concentration of perovskite (CsPbBr_2_I nanocrystals), the base color of these PhC particles changed from the off‐white of silica to the brown of CsPbBr_2_I (Figure S4a–f, Supporting Information). Compared with bare PhC microspheres, the characteristic reflection peaks of perovskite integrated PhC microspheres showed a slight red shift, which was due to the refractive index (*n*_average_) change of the microspheres as a whole (Figure S4g–i, Supporting Information). However, the presence of the reflection peak indicated that the PNs‐integrated PhC microspheres still maintained structural color, which make them ideal for serving as the coding elements in information security.

**Figure 4:**
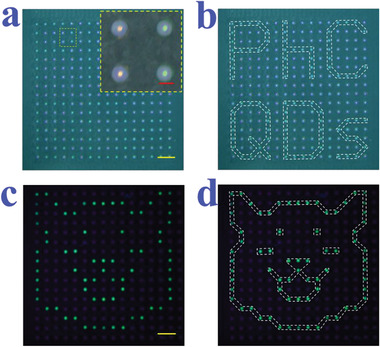
a) Optical microscopy images of the array. The inset picture in the yellow dotted line box is the enlarged view of four PhC microspheres. The red PhC microspheres are consisted of silica nanoparticles with a size of 271 nm; The green PhC microspheres are consisted of silica nanoparticles with a size of 232 nm. b) The structural color signal is “PhC, QDs.” c) Fluorescence microscopy images of the array. d) The fluorescent pattern is " Shiba Inu". The red scale bar is 300 µm and the yellow scale bars are 2 mm.

Subsequently, the fluorescence ability of PNs inside PhC microspheres has been investigated and optimized. The PhC particles were treated with different concentrations of perovskite solution, and then arranged according to the concentration gradient. Optical microscopy images showed that the base color of PhC microspheres gradually darkened with the increase of perovskite solution concentration (Figure S5a–c, Supporting Information). However, the fluorescence of PNs was not linearly correlated with its concentration (Figure S5d–f, Supporting Information), because the large size of PNs formed at high concentration resulted in aggregation‐caused quenching (ACQ) phenomenon. Thus, a concentration of 0.4 mol L^‐1^ was selected as the reaction condition. By changing the composition of perovskite solution, fluorescence signals with different color could be obtained (Figure 3b). In addition, PNs would undergo conformational changes under water‐rich conditions, resulting in the quenching of photoluminescence. The spectral data showed that the fluorescence peak of PNs‐integrated PhC microspheres disappeared (Figure 3c). In contrast, by introducing other fluorescent substances such as semiconductor quantum dots (QDs), the PhC microspheres initially showed a composite fluorescent color. When in a wet environment, the fluorescence of PNs was quenched and only the fluorescence of QDs appeared (Figure 3d). This invertible fluorescence of PN‐integrated PhC microspheres is a mimic of signal transmission in signal model of traffic light. Therefore, it is possible for the PN‐integrated PhC microspheres to be employed for anti‐counterfeiting by utilizing the dual optical signal from both PNs with invertible fluorescence and PhCs with bright structural color (**Figure** **4**).

**Figure 5:**
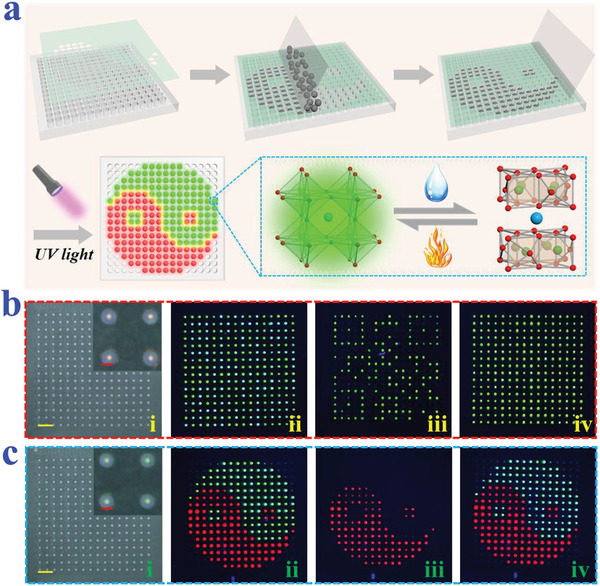
a) Schematic illustration of the preparation process and hydrochromic phenomenon of PNs‐integrated PhC microsphere arrays. b) Using the PNs‐integrated PhC microsphere arrays to encrypt a QR code: (i) Optical microscope images of the array. The inset picture is the enlarged view of four red PhC microspheres, which are consisted of silica nanoparticles with a size of 271 nm; (ii) Fluorescent microscopy images of the array; (iii) Fluorescent microscopy images of the array after wetting; (iv) Fluorescent microscopy images of the array after redrying. c) Using the PNs‐integrated PhC microsphere arrays to encrypt a Taichi pattern: (i) Optical microscopy images of the array. The inset picture is the enlarged view of four green PhC microspheres, which are consisted of silica nanoparticles with a size of 232 nm; (ii) Fluorescent microscopy images of the array; (iii) Fluorescent microscopy images of the array after wetting; (iv) Fluorescent microscopy images of the array after redrying. The red scale bar is 300 µm and the yellow scale bars are 2 mm.

In order to improve the practicability of the PNs‐integrated PhC microsphere arrays for information security, multimask superposition approach was adopted to prepare more complex patterns. First, polymethyl methacrylate (PMMA) was employed to fabricate a microcylindrical array, and then polydimethylsiloxane (PDMS) was injected into the microcylindrical array to replicate a substrate with orderly holes (Figure S6, Supporting Information). After this, the PDMS substrate was treated with fluorosilane to impart it hydrophobic at the surface. The PhC microspheres with different optical coding elements (structural color and PNs’ fluorescence) were classified and placed on corresponding masks. Then the PN‐integrated PhC microspheres were scraped into the holes of PDMS substrate according to the pattern, while the other holes were obscured by the mask (**Figure** **5**a). The metal mask was regarded as a “gate”; only the correct PhC microspheres could pass through the mask and fall into the hole in the PDMS, while the other microspheres would be blocked by the mask and eventually scraped away. The PNs inside PhC microspheres would undergo conformational changes under water‐rich conditions, resulting in the quenching of photoluminescence. The nanostructure in PNs‐integrated PhC array could lock the solution by capillary force to avoid ion's runoff, thus endowing PNs with the ability of hydrochromic reversibility after the array was dried. In addition, the sprayed moisture tends to converge on the hydrophilic PhC microspheres instead of the hydrophobic PDMS substrate, in a way similar with the wettability feature of *Stenocara*. Thus, the hydrophobic PDMS substrate effectively prevents the pollution between PhC microspheres. In contrast, if using a hydrophilic substrate to construct the photonic crystal microspheres array, the imposed moisture would spread from the microspheres onto the substrate, thus causing ion loss of the PNs. As a result, when the moisture volatilized, the PNs in the PhC microspheres could not restore their original fluorescence, resulting in mutual contamination of signals. Moreover, by combing the hydrochromic PNs and other fluorescent substances (such as semiconductor QDs) without hydrochromic phenomenon, safer information encryption could be achieved through the conversion between wet and dry environments.

To demonstrate this, PNs and QDs were respectively encapsulated into red structural color PhC particles, and then loaded into PDMS substrate to form an array (Figure 5b‐i). Under ultraviolet light, both PhC particles emitted green fluorescence, making the pattern unrecognizable (Figure 5b‐ii and Figure S7a, Supporting Information). By breathing on the PhC array to moisten it, the photoluminescence of PNs was quenched while that of QDs was retained, thus revealing the pattern of a QR code (Figure 5b‐ iii, Figure S7b, Supporting Information). After obtaining information, the photoluminescence of the PNs would be restored under the drying condition, which hid the information again (Figure 5b‐iv and Figure S7c, Supporting Information). By introducing PNs or QDs with other colors, the pattern could be designed with more complex geometry, thus improving security (Figure 5c and Figure S8, Supporting Information). It was noteworthy that PNs and QDs could be integrated in the same PhC microsphere simultaneously. Under wet conditions, the fluorescence of PNs was quenched, and PhC only showed the fluorescence signal of QDs. When the environment became dry, the fluorescence of PNs was recovered and the PhC showed composite fluorescence signal. The composite fluorescence consists of PNs’ fluorescence and QDs’ fluorescence, whose color can be adjusted by changing the ratio of these two kinds of fluorescence and the position of the emission peak.

The encrypted fluorescence pattern made by the PNs/QDs‐integrated PhC microspheres could be endowed with a high level of complexity, thus improving the information security. As shown in Figure S9a (Supporting Information), there is a house on the land as the weather is fine. When the weather turned to rainy (thus wetting the PNs‐integrated PhC microspheres array), it could be observed that a red smoke rose from the house. After the weather cleared up (thus drying the array), the smoke disappeared. Without moisture, the PhC microspheres of smoke pattern displayed a composite yellow color of red QDs and green PNs, which is almost indistinguishable from the surrounding yellow QDs (Figure S9b, Supporting Information). Breathing to moisten it, the fluorescence of PNs disappeared and only the QDs’ fluorescence left, thus exposing the red smoke pattern. After re‐drying, the fluorescence of PNs recovered and the red smoke pattern was hidden again. We next conducted cyclic wet‐dry tests to study the stability of the PNs‐integrated PhC microspheres, as shown in Figure S10 (Supporting Information). We found that the microspheres placed on the surface PDMS could maintain the fluorescence after eight cycles, while those embedded in PDMS had a slight drop of the fluorescence intensity. We suspected that this is because the cylindrical holes in PDMS do not exactly match the PNs‐integrated PhC microspheres. Therefore, part of the solution remains in the corners of the cylinder when the microspheres were infiltrated. When the PNs‐integrated PhC microspheres were dried again, the amount of PNs contained in the microspheres decreased, resulting in a slight decrease of the fluorescence intensity. These results indicated that the hydrochromic phenomenon of PNs‐integrated PhC microsphere arrays could be utilized for information encryption.

**Figure 6:**
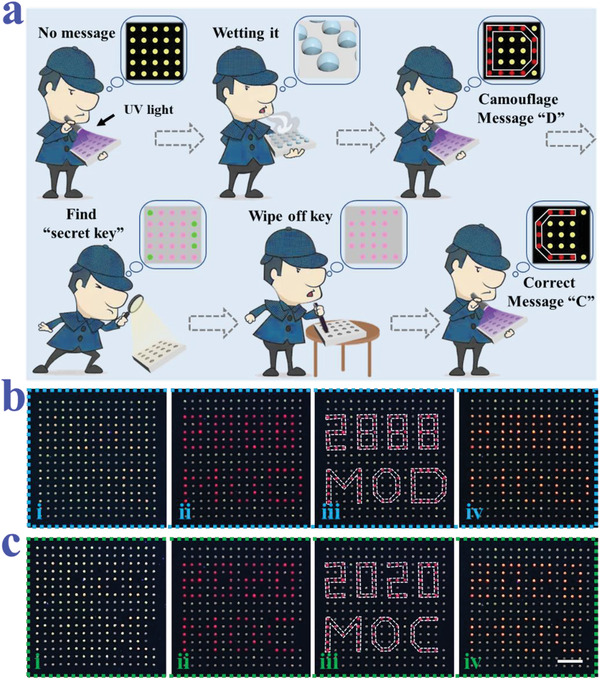
a) Schematic illustration of the information encryption process of the PNs‐integrated PhC microsphere arrays by hydrochromic process and structural color. b) The fluorescent microscope images of the array before wiping off the secret key: (i) initial state; (ii) wetting state; (iii) the hidden information; (iv) drying state. c) The fluorescent microscope images of the array after wiping off the secret key: (i) initial state; (ii) wetting state; (iii) the hidden information; (iv) drying state. Scale bar is 3 mm.

In order to develop more secure encryption strategy, we further expanded this hydrochromic property. Since the structural color signal of PhC microspheres could only be distinguished by magnified observation, which made it suitable to serve as the secret key. In some critical positions, green PhC microspheres were employed to replace the red ones. Subsequently, these PhC microspheres in the array were encapsulated with PNs and QDs to endow them with photoluminescence property. Under ultraviolet light, PhC microspheres in the array displayed yellow fluorescence of QDs, or composite yellow fluorescence of red QDs and green PNs (**Figure** **6**a). After wetting the array, it was observed that the fluorescence of PNs in some PhC microspheres was quenched, making them show red fluorescence of QDs. Then, the PhC microspheres with red fluorescence were connected to reveal its hidden message. However, the information we obtained was the camouflage message. From magnified observation, a small quality of green PhC microspheres were found mixed in the red PhC microsphere arrays as the “secret key.” We can use a suitable mask to hide the PhC microspheres and tape the green PhC microspheres away. By removing these green PhC microspheres and then repeating the above process, the correct message would be found on the array. Finally, after putting the array in the oven for drying, the message with red fluorescent was hidden again. Through this strategy, we realized the encryption and anti‐counterfeiting of the messages (Figure 6b,c and Figure S11, Supporting Information). In addition, by introducing more PhC microspheres with different structural colors, the “secret key” could also be hidden. This means that if you do not know which structural color acts as the secret key, the final message may be completely wrong. Moreover, the method of removing the specific PhC microspheres to reveal true information is a non‐reusable method, similar to a scratchcard. These features enable the PNs‐integrated PhC microsphere arrays to be used as a disposable security label. These results indicated that the integration of these two optical coding signals greatly increased the complex rate of this encryption method, which would drive the development of information security.

## 3 Conclusion

In summary, inspired by the *Stenocara* beetles and fireflies, we have developed a novel array of PN‐integrated PhC microsphere for information security. These microspheres were imparted with binary coding optical signals by angle‐independent spherical PhC and encapsulated PNs. The hydrophilic PhC microspheres were placed into the hydrophobic PDMS substrate to form a wettability structure through the multimask superposition approach. These PhC microspheres could converge moisture in wet environment to avoid the ions loss of the PNs. Besides, the PDMS substrate could effectively prevent the mutual contamination among different PhC microspheres. It was worth noting that the photoluminescence of the PNs‐integrated PhC microspheres could reversibly quench or recover in response to the moisture of environments. Based on this strategy, we realized the encryption and anti‐counterfeiting of the messages. These features indicated that the proposed PN‐integrated PhC microsphere arrays have promising prospects in message encryption, which was expected to break new ground in the field of information security.

## 4 Experimental Section

### Materials

All the reagents could be used directly in the experiments without pretreatment. The polymethyl methacrylate (PMMA) microcylindrical array was processed by Zhongxinqiheng Co., Ltd. Polydimethylsiloxane (PDMS) and its curing agent were brought from Dow Corning. Silica nanoparticles were self‐prepared using tetraethyl orthosilicate (TEOS, Sigma‐Aldrich), ammonia (Sigma‐Aldrich), and ethyl alcohol (Sigma‐Aldrich). The reagents employed to in‐situ generate the perovskite nanocrystals (PbCl_2_, PbBr_2_, PbI_2_, CsCl, CsBr, CsbI, and *N*,*N*‐dimethylformamide) were purchased from Aladdin Co., Ltd. PEG‐COOH modified semiconductor quantum dots (CdSe/ZnS, 470nm, 525nm and 625 nm) were brought from Xingshuo Nanotech Co., Ltd. (Suzhou, China). Deionized water (18.2 M cm) was obtained from the Millipore Milli‐Q system.

### Preparation of PN‐Integrated PhC Microspheres

SiO_2_ with different diameters were fabricated by oneself. A solution of 40 mL ethanol (98%) containing 1.5 mL TEOS was stirred evenly in the flask. Then 40 mL ethanol (98%) containing 10 mL ammonia was added to the flask drop by drop. After 5 h, the reaction was stopped and the SiO_2_ nanoparticles were centrifuged. By increasing the amount of TEOS, the size of the SiO_2_ nanoparticles could be increased. Then microfluidic device was employed to generate the droplets containing SiO_2_ nanoparticles. With the evaporation of water, these colloidal droplets gradually self‐assembled into photonic crystal (PhC) microspheres at 75 °C for 9 h. Subsequently, these PhC microspheres were placed in an oven at 800 °C and calcined for 8 h to slightly melt the surface of the SiO_2_ nanoparticles. After cooling, these nanoparticles connected to each other, increasing the overall mechanical strength of the PhC microspheres. DMF solutions containing perovskite were prepared by directly mixing two different DMF solutions dissolving 0.4 m PbX_2_ (X = Cl, Br, I) and 0.4 m CsX (X = Cl, Br, I), respectively. Different perovskite nanocrystals can be obtained by adjusting the composition of X (X = Cl, Br, I) element. The PhC microspheres were immersed in the perovskite solution for 10 min to fully fill the gaps. Then the PhC microspheres containing perovskite solution were brought out and put in the oven at 50 °C for 10 min to evaporate the DMF. Finally, these PN‐integrated PhC microspheres were classified by coding color.

### Preparation of the Array

PDMS and the curing agent were mixed with a ratio of 9:1 and then poured on the PMMA microcylindrical formwork. Subsequently, the formwork with PDMS was placed in the oven at 60 °C for 6 h and waited for curing. The PDMS substrate was delaminated to obtain a 16 × 16 microporous array. Then the array was treated with 2 v/v % (Heptadecafluoro‐1,1,2,2‐tetradecyl)trimethoxysilane to impart it with a hydrophobic surface. After hydrophobic treatment, the PDMS substrate was covered with a metal mask and the PN‐integrated PhC microspheres were then pushed into the hole. By replacing another mask and repeating several times to get the desired PNs‐integrated PhC microsphere arrays.

### Characterization

The microstructures and energy spectrum were characterized by a field emission scanning electron microscopy (FESEM, Hitachi, S‐300N). The fluorescent images of PNs‐integrated PhC microspheres were obtained by a fluorescence microscope (Olympus, CKX41) equipped with a CCD camera (Olympus, DP30BW). The reflection spectra and fluorescent intensity were measured by the same microscope equipped with an optic spectrometer (Ocean Optics, USB 2000+). The fluorescent images of the array were obtained by the mobile phone (Honor, 20pro).

## Conflict of Interest

The authors declare no conflict of interest.

## Supporting information

Supporting Information

## Data Availability

The data that support the findings of this study are available in the supplementary material of this article.
