# Supplementary material for: Bioinspired Perovskite Nanocrystals‐Integrated Photonic Crystal Microsphere Arrays for Information Security
Source: Adv Sci (Weinh). 2022 Jan 20;9(9):2105278. doi: 10.1002/advs.202105278 (PMC8948562; doi:10.1002/advs.202105278)
Supplement: Supplementary file 1 — Supporting Information [file ADVS-9-2105278-s001.pdf]

ADVANCED SCIENCE
Open Access

Supporting Information

for *Adv. Sci.*, DOI 10.1002/advs.202105278

Bioinspired Perovskite Nanocrystals-Integrated Photonic Crystal Microsphere Arrays for Information Security

Feika Bian, Lingyu Sun, Hanxu Chen, Yu Wang, Li Wang, Luoran Shang and Yuanjin Zhao**

WILEY-VCH

ADVANCED SCIENCE
Open Access

Supporting Information

for *Adv. Sci.*, DOI: 10.1002/advs.202105278

Bioinspired perovskite nanocrystals-integrated photonic crystal microsphere arrays for information security

Feika Bian, Lingyu Sun, Hanxu Chen, Yu Wang, Li Wang, Luoran Shang, and Yuanjin Zhao**

((Supporting Information can be included here using this template))

© 2021 Wiley-VCH GmbH

Supporting Information

Bioinspired perovskite nanocrystals-integrated photonic crystal microsphere arrays for information security

Feika Bian [a], *Lingyu Sun* [b], *Hanxu Chen* [b], *Yu Wang* [a], *Li Wang* [b], *Luoran Shang* [c,*],*Yuanjin Zhao* [a, b, *]

Dr. F. K. Bian, Dr. Y. Wang, Prof. Y. J. Zhao
[a] Department of Clinical Laboratory, Institute of Translational Medicine, The Affiliated Drum Tower Hospital of Nanjing University Medical School, 210008 Nanjing, China
E-mail: yjzhao@seu.edu.cn

Dr. L. Y. Sun, H. X. Chen, L. Wang, Prof. Y. J. Zhao
[b] State Key Laboratory of Bioelectronics, School of Biological Science and Medical Engineering, Southeast University, Nanjing 210096, China

Prof. L. R. Shang
[c] Shanghai Xuhui Central Hospital, Zhongshan-Xuhui Hospital, and the Shanghai Key Laboratory of Medical Epigenetics, International Co-laboratory of Medical Epigenetics and Metabolism (Ministry of Science and Technology, Institutes of Biomedical Sciences), Fudan University, Shanghai, China.
E-mail: luoranshang@fudan.edu.cn

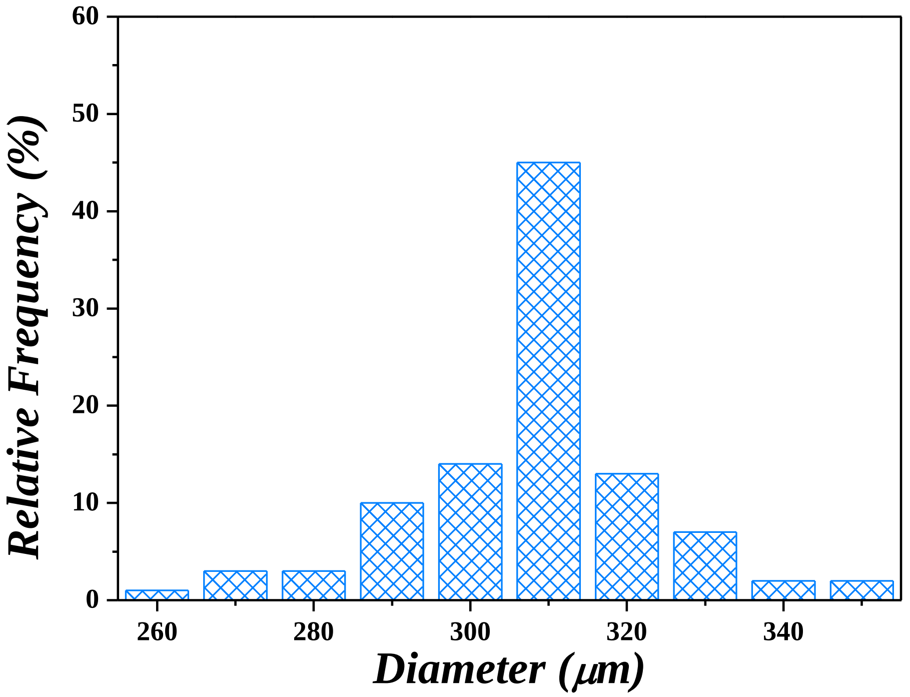

Figure S1 The size distribution of the PhC microspheres.

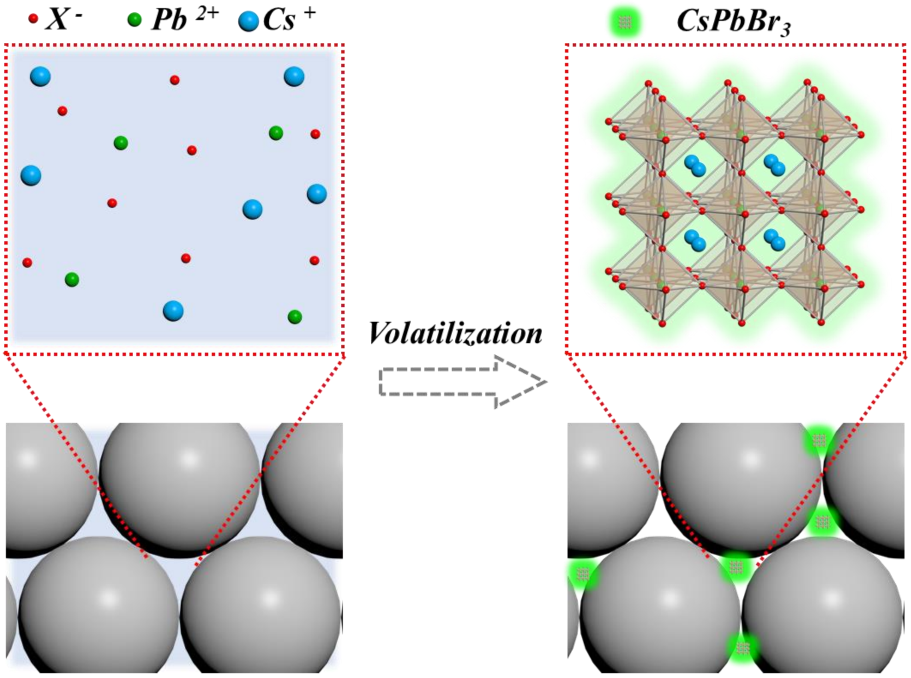

Figure S2 Schematic illustration of the PNs generated in PhCs' gaps.

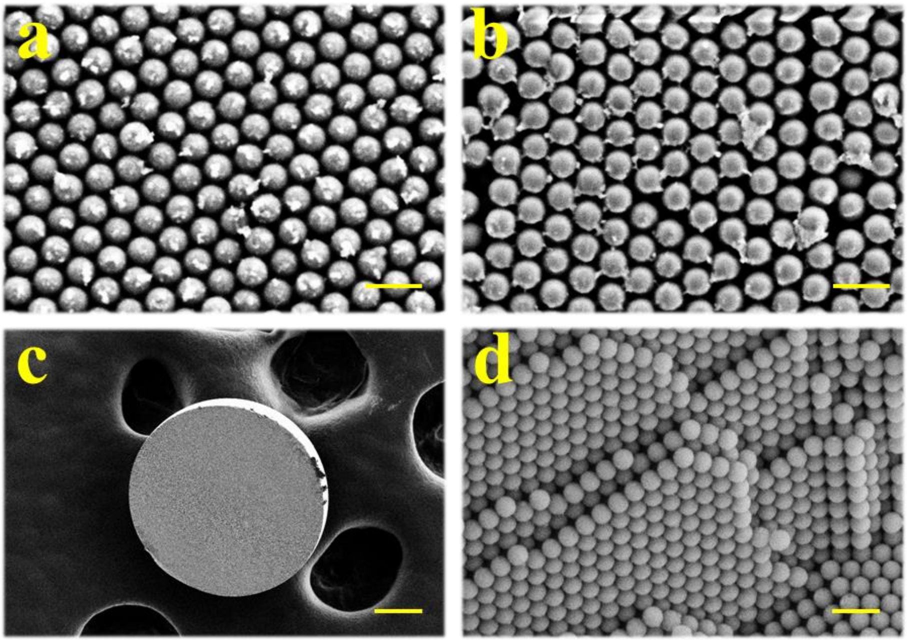

Figure S3 FESEM images of (a) PhC microsphere after decorating with 0.2 M $CsPbBr_2I$, scale bar is 500 nm; (b) PhC microsphere after decorating with 0.4 M $CsPbBr_2I$, scale bar is 500 nm; (c) PhC microsphere after decorating with 0.1 M $CsPbBr_2I$ that have been cut open, scale bar is 800 μm; (d) cross-section image showing the three-dimensional ordered closely packed structures of PhC microspheres, scale bar is 600 nm.

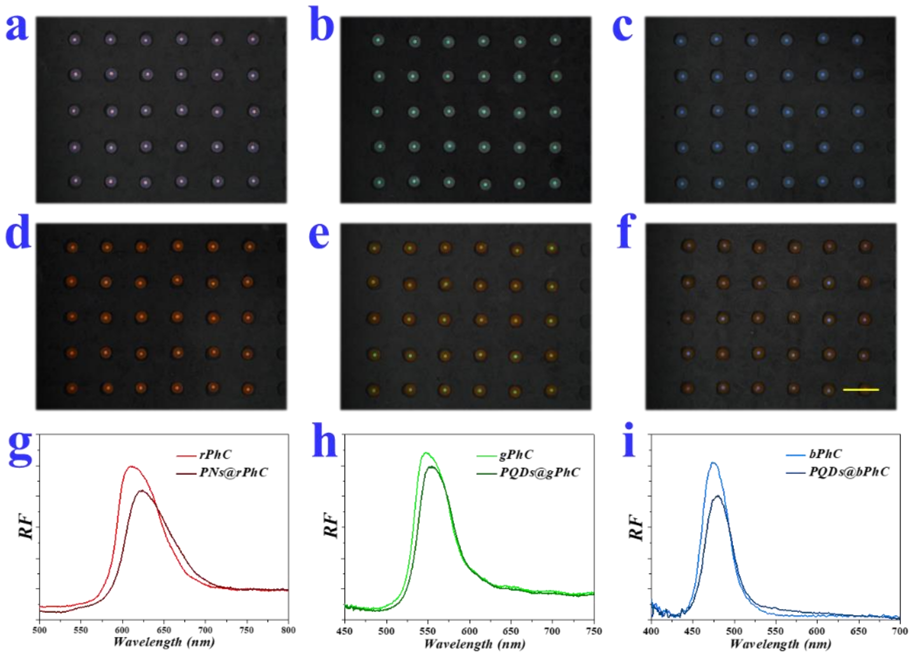

Figure S4 (a-c) Optical images of three kinds of PhC microspheres before decorating PNs. (d-f) Optical images of three kinds of PhC microspheres after decorating PNs. (g-i) Reflection spectra of three kinds of PhC microspheres before and after decorating PNs. Scale bar is 1 mm.

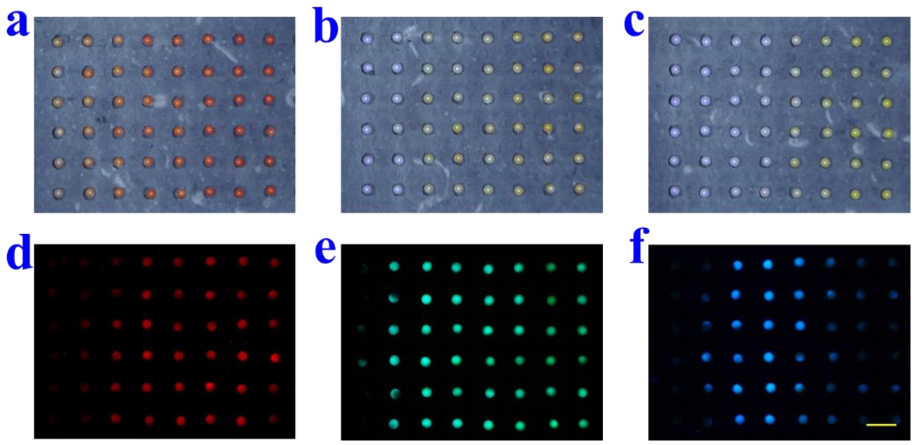

Figure S5 (a-c) Optical images of PhC microspheres after decorating three kinds of PNs with different concentrations. (d-f) Fluorescent images of PhC microspheres after decorating three kinds of PNs with different concentrations. Scale bar is 1 mm.

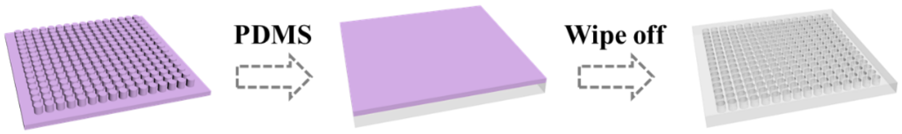

Figure S6 Schematic illustration of the preparation process of the hydrophobic PDMS substrate.

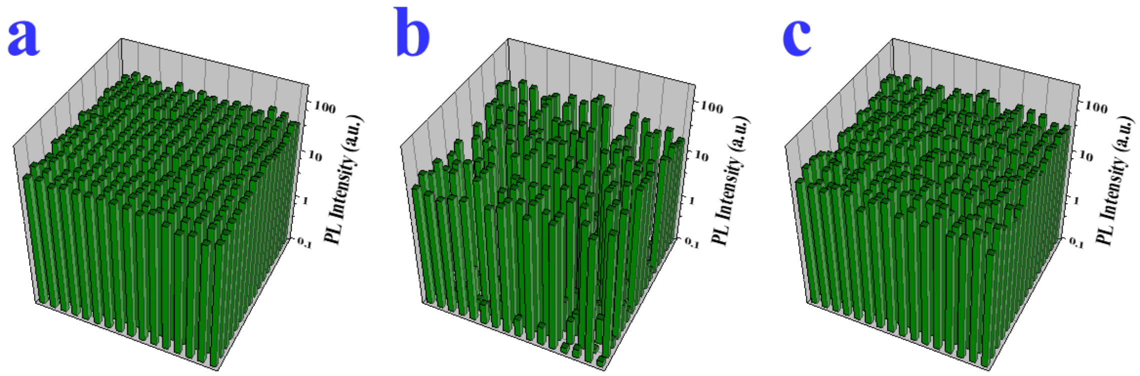

Figure S7 The fluorescence intensity of each microsphere in the QR code: (a) before wetting (a) after wetting; (c) after redrying. Green pillars refer to the fluorescence intensity of green PNs and green QDs, where the fluorescence of QDs did not change.

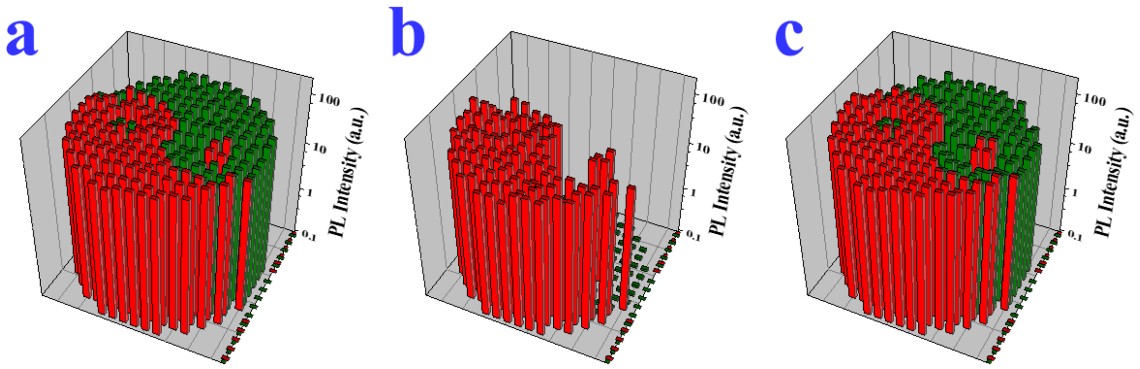

Figure S8 The fluorescence intensity of each microsphere in the Taichi pattern: (a) before wetting (a) after wetting; (c) after redrying. Green pillars refer to the fluorescence intensity of green PNs and red pillars refer to the fluorescence intensity of red QDs, where the fluorescence of QDs did not change.

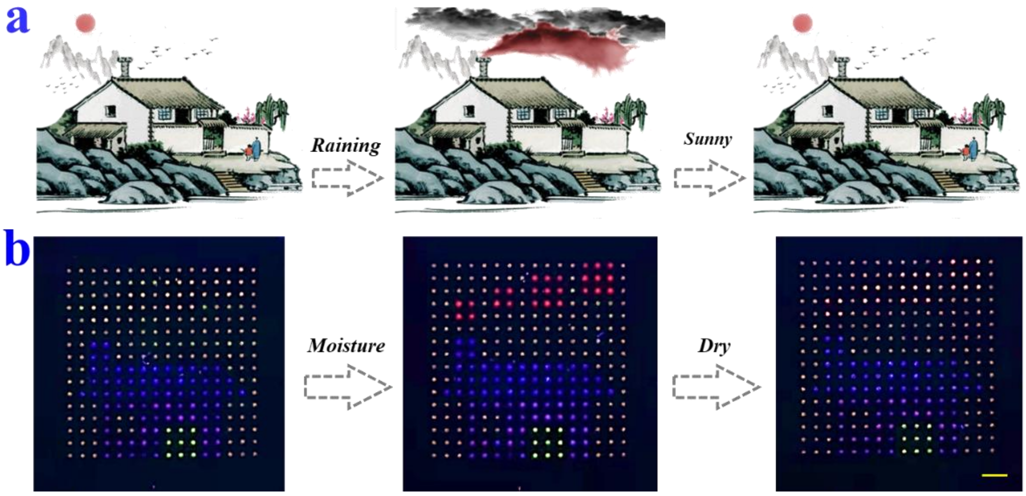

Figure S9 (a) Schematic illustration of the hydrochromic phenomenon of PNs-integrated PhC microsphere arrays. (b) Using the PNs-integrated PhC microsphere arrays to encrypt a “smoke” pattern. Scale bar is 2 mm.

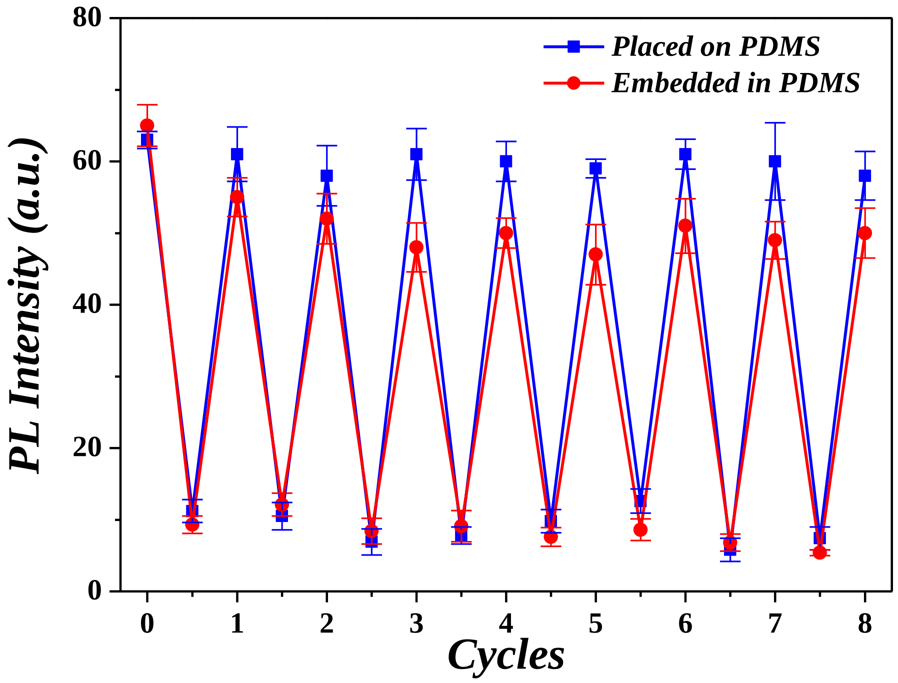

Figure S10 The wet-dry cycling stability of the PNs-integrated PhC microspheres embedded in (red) or placed on (blue) PDMS substrate.

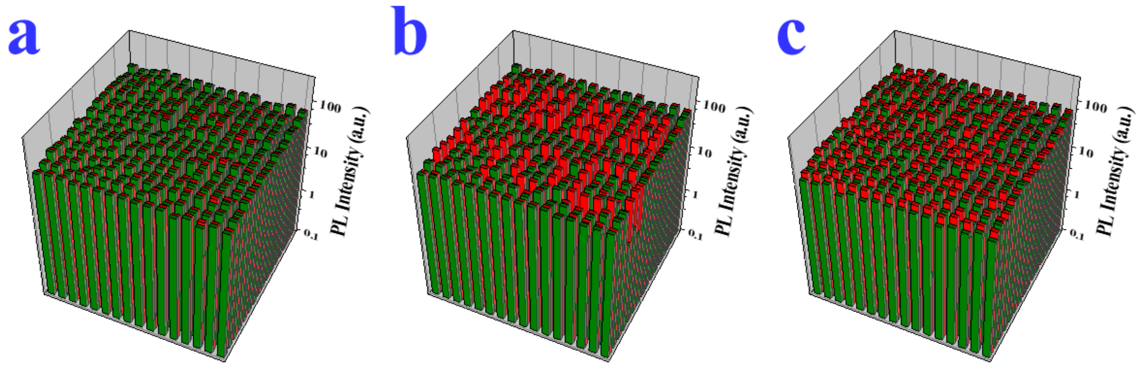

Figure S11 The fluorescence intensity of each microsphere in the information encryption pattern: (a) before wetting (a) after wetting; (c) after redrying. Red pillars refer to the fluorescence intensity of red QDs. Green pillars refer to the fluorescence intensity of green PNs and green QDs, where the fluorescence of QDs did not change.
